# Understanding the barriers to successful adoption and use of a mobile health information system in a community health center in São Paulo, Brazil: a cohort study

**DOI:** 10.1186/s12911-016-0385-1

**Published:** 2016-11-17

**Authors:** Jayant V. Rajan, Juliana Moura, Gato Gourley, Karina Kiso, Alexandre Sizilio, Ana Maria Cortez, Lee W. Riley, Maria Amelia Veras, Urmimala Sarkar

**Affiliations:** 1 Department of Medicine, San Francisco General Hospital, University of California, San Francisco, 1001 Potrero Avenue, San Francisco, CA 94110 USA; 2 Faculdade de Ciências Médicas da Santa Casa de São Paulo, Rua Dr. Cesário Motta Jr., 61, Vila Buarque, São Paulo, SP Brazil; 3 Center for Vulnerable Populations, San Francisco General Hospital, University of California, 1001 Potrero Avenue, San Francisco, CA 94110 USA; 4 School of Public Health, 530 E Li Ka Shing Center, University of California, Berkeley, CA 94720 USA

**Keywords:** mHealth, Brazil, Usability

## Abstract

**Background:**

Mobile technology to support community health has surged in popularity, yet few studies have systematically examined usability of mobile platforms for this setting.

**Methods:**

We conducted a mixed-methods study of 14 community healthcare workers at a public healthcare clinic in São Paulo, Brazil. We held focus groups with community healthcare workers to elicit their ideas about a mobile health application and used this input to build a prototype app. A pre-use test survey was administered to all participants, who subsequently use-tested the app on three different devices (iPhone, iPad mini, iPad Air). Usability was assessed by objectively scored data entry errors and through a post-use focus group held to gather open-ended feedback on end-user satisfaction.

**Results:**

All of the participants were women, ranging from 18–64 years old. A large percentage (85.7%) of participants had at least a high school education. Internet (92.8%), computer (85.7%) and cell phone (71.4%) use rates were high. Data entry error rates were also high, particularly in free text fields, ranging from 92.3 to 100%. Error rates were comparable across device type. In a post-use focus group, participants reported that they found the app easy to use and felt that its design was consistent with their vision. The participants raised several concerns, including that they did not find filling out the forms in the app to be a useful task. They also were concerned about an app potentially creating more work for them and personal security issues related to carrying a mobile device in low-income areas.

**Conclusion:**

In a cohort of formally educated community healthcare workers with high levels of personal computer and cell phone use, we identified no technological barriers to adapting their existing work to a mobile device based system. Transferring current data entry work into a mobile platform, however, uncovered underlying dissatisfaction with some data entry tasks. This dissatisfaction may be a more significant barrier than the data entry errors our testing revealed. Our results highlight the fact that without a deep understanding of local process to optimize usability, technology-based solutions in health may fail. Developing such an understanding must be a central component in the design of any mHealth solution in global health.

## Background

Wide dissemination of consumer market smartphones is a recent development that has been paralleled by the growth of wireless networks. These two developments have enabled the rapid growth of mobile health (mHealth). mHealth is of particular interest in the developing world, where it can potentially extend the reach and capacity of overburdened healthcare systems [1]. There is a growing body of mHealth literature. Text messaging has been studied extensively in the treatment of chronic conditions, including hypertension [2–4], diabetes [5–10]. The use of mobile personal health records (mPHRs) is another commonly examined mHealth application [11].

There is an urgent need to systematically determine what mHealth approaches truly improve patient care [1, 12–18]. Brazil is an ideal place to do so as it is one of the leading global emerging economies commonly referred to as BRICS (Brazil, Russia, India, China, South Africa). It has high rates of cell phone and internet use, with 135 mobile subscriptions/100 persons and 52 internet users/100 persons in 2013. These figures compare to 89/100 and 46/100 in China, 71/100 and 15/100 in India, 153/100 and 61/100 in Russia, and 146/100 and 49/100 in South Africa [19]. Setting Brazil apart from the other BRICS nations, however, is the fact that it guarantees universal access to healthcare to all of its citizens.

The Brazilian national healthcare system, known as the *Sistema Único de Saúde* (SUS), was founded in 1988 and is accessible to all Brazilians [20, 21]. The primary strategy for providing care in the SUS is the Estratégia Saúde da Família (family health strategy), the core of which is the *Equipe de Saúde da Família* (family health team; ESF). Each ESF consists of a doctor, nurse, and 4–6 community healthcare workers all of whom work together to care for a group of patients. This existing care infrastructure, Brazil’s strong telecom infrastructure and a large lower to lower middle class population that relies on the SUS are all features that make Brazil an attractive place to study mHealth.

Multiple mHealth projects have already been conducted in Brazil, including in the Western region of São Paulo [22]. In addition, the goal of a recent initiative by the Brazilian Health Ministry, e-SUS, is to provide free software to encourage adoption of electronic medical records in SUS clinics [23]. One of the modules available through the latter initiative is a mobile data collection app that seeks to replace pen and paper with a tablet. Each of these important projects and initiatives has focused either on short-term clinical interventions or surveillance. To our knowledge, prior mHealth studies in Brazil, have not specifically assessed the question of usability. In this study, we engaged with community healthcare workers (CHW), often a focal point of mHealth projects. We focused on two primary usability outcomes: data entry accuracy and end-user satisfaction [24, 25]. To better evaluate end-user satisfaction and identify additional barriers to usability we conducted a focus group with CHWs [26]. Our goal was to understand the needs of CHW and to develop a prototype app, believing that it was first necessary to understand process in order to develop a sustainable solution.

## Methods

### Site and participant description

The Centro de Saúde Escola Barra Funda (CSEBF) is a clinic located in the Western region of São Paulo. It is one of São Paulo’s many public health clinics but is unique in that it has a longstanding history of being connected to one of the city’s oldest medical schools, Santa Casa São Paulo School of Medical Sciences.

The clinic has a total of 3 ESF teams, staffed by a total of 3 physicians, 3 nurses, 6 medical assistants (*auxiliar de enfermagem*) and 18 CHW. The clinic serves a socioeconomically diverse area, ranging from a *favela* (urban slum) to middle and upper middle class areas. By law in Brazil, CHW are residents of the communities they serve. Two of their primary duties are to register everyone in the coverage area they serve, and to collect basic public health information on them for resource allocation. CHWs fill out paper forms which others manually enter into a database whose contents are sent to the state and subsequently national health ministry for surveillance purposes. CHWs’ other primary function is as intermediaries between health care providers in the clinic and community members. They are the clinic’s ‘eyes and ears’ in the community.

### Recruitment

Using convenience sampling 14 CHWs from the clinic were recruited to participate in the study. Their participation included a design focus groups, a baseline assessment survey, usability assessment, and follow up focus group. Clinic nurses and medical assistants did not participate in the study. Two of the clinic’s physicians (JM, AC) participated in facilitating the focus groups. We obtained informed consent from all participants. All data collected were de-identified, thus signatures were not obtained for consent forms. No identifying information or protected health information was obtained or discussed during the focus groups.

### Study review

The study protocol was reviewed and approved by both the University of California San Francisco (UCSF) Institutional Review Board (deemed exempt) and the Santa Casa Medical School Institutional Review Board.

### Use-case

We chose to build an app for demographic data entry because it would: (1) replace a pen-and-paper task (2) automate data entry to enhance timeliness/usability of this data (3) ubiquity of task.

### Software development and design focus group

The prototype application was developed by JR for iOS 7 using XCode 5. The application was developed utilizing an agile process. No requirements engineering or software modeling tool for this prototype development since this was not an attempt to scale the application to production [27]. A focus group was held with CSEBF’s CHWs. It lasted for 2 h and was open-ended with a semi-structured interview guide, facilitated by two of the authors (JM, AC) who are also physicians at the clinic. The focus group was not recorded, but the facilitators did take notes.

All 14 CHW participated in the design focus group. The facilitators explained the team’s research plans and elicited the CHWs’ ideas about what a prototype app would look like. CHWs were posed two primary questions: “What would be the app of your dreams?” and “What would it do and how would it look?”.

As part of this process the CHWs were divided into 3 teams (team 1 with 5 people, team 2 with 5 people, team 3 with 4 people) to develop mock-ups of the ‘app of their dreams’ detailing what its interface might look like as well as what its functions would be.

These notes and designs were provided to JR and used as templates for development of the prototype app which was later tested by the same CHWs who provided the original design input.

### Administration of pre-use survey

The same CHWs who participated in the design focus groups selected a pseudonym, unknown to the study authors that they used throughout the study. Prior to interacting with the prototype app all 14 CHWs filled out a pre-use survey. This survey was administered one day prior to the collection of test-use data and consisted of a subset of questions from the Pew internet use survey [28]. All questions were translated from English to Portuguese as well as back-translated from Portuguese to English. All collected data were entered into a Microsoft Excel spreadsheet to facilitate analysis.

### Collection of test-use data and assessment of data entry accuracy

Clinic physicians developed 3 short clinical vignettes that were used for data entry (Appendix 1). No instruction was provided to CHW about how to use the app, other than to provide them with a non-identifiable login that was not linked to their demographic information. All 14 CHWs worked in pairs, with one CHW functioning as the ‘patient’, using the vignette and the other functioning in their usual role of CHW, entering data. All CHW used three different devices: an iPod touch, an iPad Mini and an iPad Air. Each CHW entered each of the 3 simulated patients. For each CHW, the vignettes were randomly assigned to the three different devices. This group usability assessment was not audio recorded, although facilitators (JR, JM, AC) did take notes. We choose focus groups to allow for interactions between participants that can surface issues/concerns that they may be less likely to voice independently. We thought they might feel more comfortable expressing usability challenges if peers concurred. Participant burden was judged to be lower as well for focus groups, than a written questionnaire by local experts.

The data collected in the app were based on two forms produced by the SUS that are routinely used by CHW, one for diabetes and one for hypertension. All of the data fields on these forms were not represented in the clinical vignettes, which were deliberately kept brief given that each CHW had to enter data on 3 devices. Fields represented in the vignettes included: first name, last name, gender, age, smoking status, drinking status, date of visit (for both conditions), coordination of medications at home (for both conditions), and insulin use. Each of these data entry fields fell into one of three categories: 1) free text, 2) switch (on/off), 3) slider (used for age only).

For free text fields (non-date), errors were scored as: −1 for no data, 0 for no error, 1 for a spelling error, 2 for a capitalization error, 3 for a combination of spelling and capitalization, 4 for data entered in the wrong field, 5 for a combination of the other errors, 6 for any other type of error. For free text fields that represented a date, errors were scored as: −1 for no data, 0 for no error, 7 for an incorrect date, 8 for a date formatting error, 9 for any other error. For switch fields errors were scored as: 0 for no error, 1 for an error. Missing data was not possible for these fields, since their default value was ‘off’. For the single slider field, entered data was scored as in error if it did not correspond to the age given in the vignette. At present, no uniform system for scoring errors on user interfaces exists, though the method used here is consistent with work done by other human-computer interaction groups [29]. We focused on transcription errors, a measure of effectiveness [30]. Each CHW was provided with a non-identifiable login that was not linked to their demographic information.

All entered data was stored on the device, retrieved and archived by screen capture. Our process is in line with previous descriptions of mHealth tracking app development [31, 32].

### Post use focus-group

One day after the data-entry exercise, we held a 2 h focus group with 12/14 CHW participants that filled out the pre-use survey [33–35]. Two CHWs were not able to attend as they did not come to the office that day for unspecified reasons. The purpose of this group was to elicit CHW’s reactions to the software, their satisfaction with the process and to have them identify any future barriers to use. The proceedings of the focus group were audio recorded and detailed notes taken and summarized. No identifying information was recorded. CHWs sat together in a private room, with facilitators leading discussion using a semi-structured interview guide. The purpose of the focus groups was to obtain feedback from CHWs about the apps usability, their preference or dislike for the app. There were open ended questions intended to elicit discussion among the CHWs. The app prototypes that were developed in pre-app focus groups were revisited and compared to their test with the developed app. Specific questions included, “what do you think about the value and use of the app?” Facilitators followed up on issues raised by CHWs in order to obtain additional detail about the CHWs thoughts.

## Results

### Participant demographics

All of the 14 participants who filled out the pre-use survey were women. They ranged in age from 18 to 64 years, with 50% between the ages of 35 and 49. A majority of CHW (12/14, 85.7%) had a middle or high school level education.

### App development

**Fig. 1:**
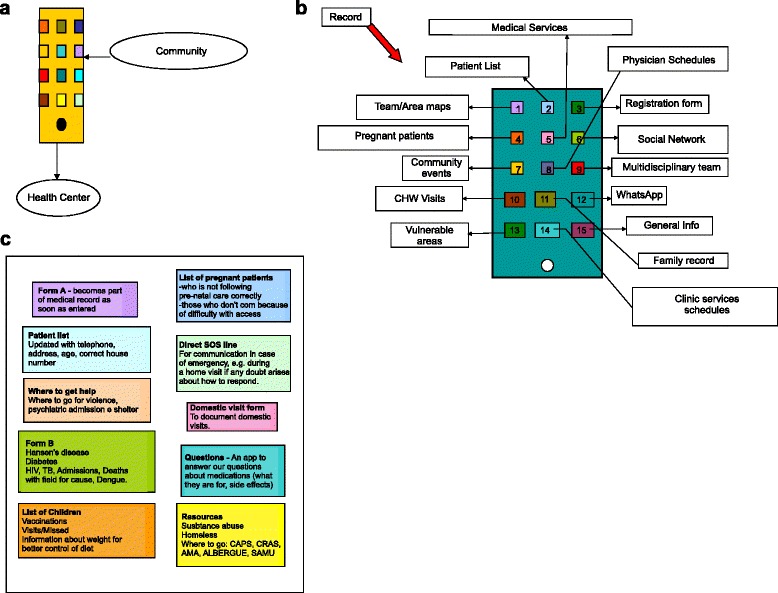
Application prototypes developed by community healthcare worker small groups. Before the prototype application was built, focus groups were held during which community healthcare workers were asked what their ideal application would look like and what functions it should have. The community healthcare workers were partitioned into three groups and the designs shown here are what they produced (﻿**a**-**c**). The prototype app was built based off of these designs

**Fig. 2:**
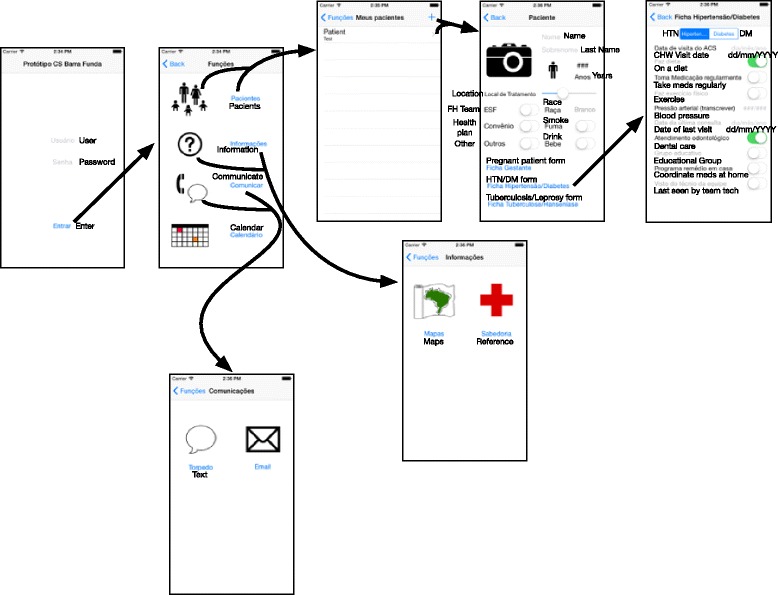
Schematic of App flow. Design of the functioning prototype app was based on the designs developed by community healthcare workers shown in Fig. 1. The flow of the prototype app is shown here for the phone version of the app. The tablet version (not shown) is identical. Screen shots shown here were taken from the iOS simulator running on Xcode 5

Figure 1 shows the three app ‘mock-ups’ produced by the CHW. Although each of these designs were different, they shared several common features: 1) an emphasis on streamlining required data entry, 2) access to relevant health information (e.g. clinic schedules) in the field, 3) the importance of integrating any collected data into the existing data management system, 4) real time communication with the clinic/clinic staff. The prototype app was developed based on the CHWs’ designs. Figure 2 shows screenshots of the app, depicting its flow.

### Pre-use survey results

**Fig. 3:**
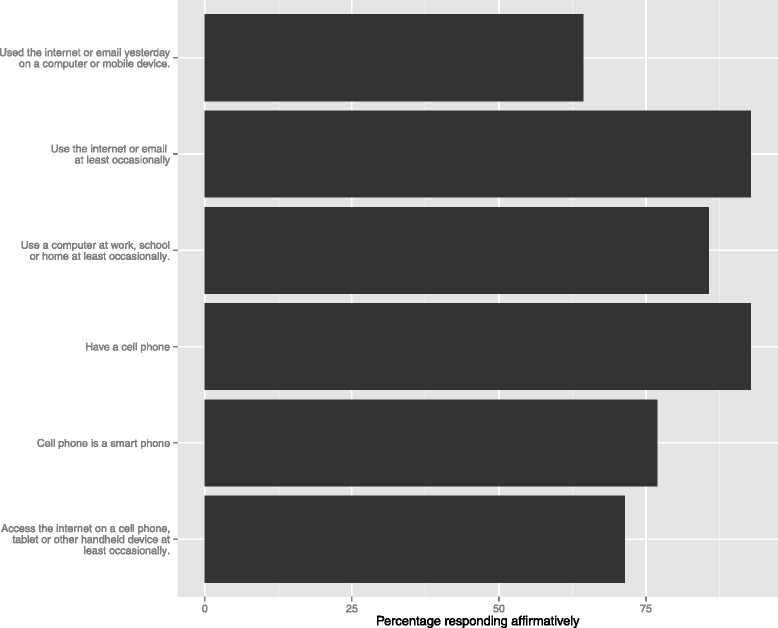
Results of Pew Internet Use Survey. All participants responded to a subset of the Pew Internet Use Survey questionnaire. Bars show the proportion responding affirmatively to the questions indicated on the graph’s y-axis

Figure 3 shows a subset of the results of the pre-use survey. 85.7% of CHW used a computer at work, school or at home and 92.8% used the internet or email. 92.8% of the participants owned a cell phone and 71.4% used the internet on a phone or other mobile device. Of those who had a cell phone, 76.9% reported having a smart phone. 64.3% of participants reported using the internet within a day and 100% of those who did, did so at home. 42.8% of participants reported that it would be difficult for them to give up using the internet. The reasons given for this difficulty varied: 16.7% reported that the internet use was essential for professional or other reasons, 16.7% reported that it was a question of enjoyment, 33.3% reported that it was a combination of both, and 33.3% reported that it was for neither of these reasons. 28.5% of participants reported that they had started using the internet between 2000–2004, 14.3% between 2005–2009, 35.7% between 2010–2014, 7.2% or as long as they could remember, and 14.3% for an unknown amount of time.

### Error scoring of data entry

Table 1 shows the results of error scoring of the data entered by participants. For the three types of data entry fields, free-text, switch and slider, we did not observe large differences in the error rate by device type. Error rates for switch-type fields were generally lower compared to those for free text fields, ranging from 0% to 7.7%. The switch-type fields where participants registered whether the patient managed their medications at home had higher error rates. For diabetes medication management, these error rates ranged from 30.8-41.8% and for hypertension they ranged from 41.7% to 69.2%.

**Table 1:** Summary of error rates and classification of error types by device

Field	Type	Device	Overall error rate (frequency, %)	Most common error type (frequency, %)
Correct Patient	N/A	Phone	1/13 (7.7)	--
		Mini	0/13 (0)	--
		Air	3/12 (25)	--
Gender	Switch	Phone	0/13 (0)	--
		Mini	0/13 (0)	--
		Air	0/12 (0)	--
Age	Slider	Phone	0/13 (0)	--
		Mini	0/13 (0)	--
		Air	2/12 (16.7)	Other (2/2, 100)
Smoking	Switch	Phone	0/13 (0)	--
		Mini	0/13 (0)	--
		Air	0/12 (0)	--
Drinking	Switch	Phone	1/13 (7.7)	Other (2/2, 100)
		Mini	0/13 (0)	--
		Air	0/12 (0)	--
First Name	Free Text	Phone	12/13 (92.3)	Grammar/Formatting (12/12, 100)
		Mini	12/13 (92.3)	Grammar/Formatting (12/12, 100)
		Air	11/12 (91.7)	Grammar/Formatting (10/11, 90.9)
Last Name	Free Text	Phone	12/13 (92.3)	Grammar/Formatting (12/12, 100)
		Mini	13/13 (100)	Grammar/Formatting (13/13, 100)
		Air	11/12 (91.7)	Grammar/Formatting (10/11, 90.9%)
Date last seen for diabetes	Free Text	Phone	13/13 (100)	Missing (12/13, 92.3)
		Mini	10/13 (76.9)	Missing (9/10, 90)
		Air	10/12 (83.3)	Missing (9/10, 90)
Date last seen for hypertension	Free Text	Phone	7/13 (53.8)	Missing (7/7, 100)
		Mini	7/13 (53.8)	Other (6/7, 85.7)
		Air	5/12 (41.7)	Missing (3/5, 60.0)
Blood Pressure	Free Text	Phone	4/13 (30.8)	Other (4/4, 100)
		Mini	2/13 (15.4)	Other (2/2, 100)
		Air	5/12 (41.7)	Other (5/5, 100)
Take insulin	Switch	Phone	0/13 (0)	--
		Mini	1/13 (7.7)	--
		Air	0/12 (0)	--
Manage diabetes medications	Switch	Phone	4/13 (30.8)	--
		Mini	6/13 (46.1)	--
		Air	4/13 (30.8)	--
Manage hypertension medications	Switch	Phone	7/13 (53.8)	--
		Mini	9/13 (69.2)	--
		Air	5/12 (41.7)	--

Error rates for free text fields were in general higher. For the two name fields (first, last), error rates ranged from 92.3%-100%. The majority of these errors across devices (90.9-100%) were grammatical and/or spelling errors in free-text fields. Error rates for the two visit date fields were higher for date of the last diabetes visit versus last hypertension visit (in both cases, the date was intended to be the date of the exercise). For diabetes visits, the error rate ranged from 76.9-100%, with 90–92.3% of these errors being due to missing (not entered) data. Error rates for hypertension were lower, ranging from 41.7%-53.8%. On the iPhone 100% of these errors were due to missing data. On the iPad Air, 60% were due to missing data, 20% were formatting errors, and 20% were classified other (other data entered, e.g. medication name). On the iPad Mini, 14.3% of errors were due to missing data, while 85.7% were classified as other and again predominantly represented other types of data (again primarily medication names).

### Post-use focus group

One day after the data entry exercise, a focus group was held with CHW. They were asked to give their impressions of the prototype app and its usability, their thoughts on the process that led to the creation of the app, and any potential barriers to its use/adoption. Overall, CHW found the app easy to use/understand and did feel that its use was concordant with their vision. Even so, several important points arose from this discussion. The first point was the unpopularity of the paper forms they are required to fill out. The value of these data (used for surveillance) was not evident to CHW, and therefore, they did not prioritize collecting complete and accurate data.

*‘aí parece que falhamos porque os pacientes tem uma postura com os agentes e outra com os homens de branco’.*

*“So it looks like we’re lacking because patients behave one way with us and another way with ‘the men in white’ (physicians).”*

A second point was that many of the CHW used a notebook in addition to the required forms to record information about patients that they did not want to put in an official record.

*‘tudo é no caderno’*

*“everything is in the notebook”*

Some said that even if they had a device that made data entry easy, they would still continue to use these notebooks.

Along these lines, there was some concern that the app could result in creating more work.

*‘tenho raiva do campo domicilio porque não é o que a gente vê’*

*“The ‘home’ field makes me angry, because it doesn’t reflect what we see”*

*‘não entendi se era para visita ou cadastro, ficou confuso’*

*“I didn’t understand if it was for a normal visit or just to register people – I was confused”*

Finally, CHW raised concerns about personal security when using a costly device in low-income urban areas, expressing a preference for a smaller device which could more easily be concealed on their persons.

## Discussion

The intersection of mHealth and global health is a rapidly growing sector, in part because of its potential to extend healthcare system capacity in resource poor or resource-limited settings. In an all-female cohort of CHW based at an academic-medical center associated clinic in São Paulo, Brazil, we observed high cell phone (92.8%), computer (85.7%), internet (71.4%) and smart-phone use (76.9%) use among CHW. We assessed usability by examining two specific outcomes: data entry accuracy and end-user satisfaction. Despite having a relatively well-educated cohort, we observed substantial data entry error. Error rates were highest in free-text fields and unrelated to device type. We also identified issues with end-user satisfaction through a CHW focus group. Implementing the mobile platform raised concerns about CHWs’ assigned tasks, with CHW reporting limited buy-in to and satisfaction with the current data entry burden of their work. The focus group also identified concerns about sensitive information (e.g. domestic violence), which CHW were hesitant to include in an official record as well as concerns about becoming a target of theft by carrying around an expensive electronic device.

The fact that our CHW cohort was all female is unsurprising. A 2007 report indicated 101,307 of 129,763 CHW in all of Brazil were female (78%) [23]. The high rates of computer and cell phone use we observed suggest that device/app uptake is unlikely to be a barrier in our study population. CHW were able to use our app with no instruction, reflecting both their education levels as well as their input in the design of the user interface. Prior studies in Africa and Latin America have reported positive perceptions of mHealth initiatives by CHW, but that this perception did not always predict effective uptake [12, 16]. One potential explanation for these results is a lack of early engagement of end-users. Early, active engagement can have significant benefits: it can identify functionality that end-users consider essential as well as potential barriers to technology adoption. In this study, for example, we identified a process that the CHW use (taking of notes in private notebooks to record sensitive information that the patient may not want in the official form) as well as CHW skepticism about the utility of collecting demographic data. If not accounted for, both of these factors could hamper app adoption/use. The process of introducing a new technology can thus uncover existing workflow/process problems which directly affect end-user satisfaction. Regardless of the ease of use of an app, without addressing such barriers, no app is likely to be used effectively.

Despite the high rates of computer and cell phone use in our study population, data entry error was a persistent problem. The highest error rates were in free-text entry fields across all device types, highlighting the importance of designing data entry strategies that minimize typing, as free-text data entry is known to create errors [36]. Possible alternatives include voice and/or handwriting recognition, neither of which is error-free [37, 38]. As the mobile health sector continues to grow, the development of novel data entry technologies that improve both the efficiency and fidelity of data entry needs to be a priority. Another potential solution is the incorporation of automated data evaluation at the back-end. In the case of text fields, for example, data quality filters using natural language processing could be useful. Implementing filters for non-text fields such as switches is more complicated, as it is necessary to distinguish between a true negative response and a non-response. The latter issue is magnified by electronic data entry, although it can also occur with paper forms. A combination of intelligent interface design and metadata (e.g. requiring that a field be actively turned on and retaining this information as metadata) could address both of these issues for non-text fields. Regardless, these points draw attention to a central point: electronic data entry results in less data entry error but does not solve data fidelity issues, a point recently illustrated in a study directly comparing paper data entry to electronic data entry [13]. Future study will be required to look at reasons for the difference in error rates for hypertension versus diabetes visit date. Possible reasons could be the need for clearer instruction in the vignettes, the placement of fields in the app, and differences in form factor.

The primary limitation of our work is its generalizability and limited external validity. Brazil has a well-structured and well-established public health system with relatively well-educated CHWs. Whether results from CHW end-users in Brazil can be extrapolated to other less well-resourced contexts is unclear. Even within Brazil, the public health sector in São Paulo is arguably both better-funded and administered than in other parts of the country. Therefore, whether the results from a single, academic medical center-connected clinic in São Paulo will apply to other less-connected clinics even in the same city is unclear. Recognizing these limitations, we nevertheless believe that our results are encouraging and an important addition to the growing body of mHealth literature. Another important limitation is that this formative study does not have the power to establish accuracy with statistical testing; we plan to pursue that with our next, larger iteration. We would also have preferred to have each participant test on all 3 devices, but in order to limit participant burden we limited the amount of testing per participant. Each vignette contained similar data for the CHWs to enter, meaning there was not much deviation in entry required across vignettes. An additional limitation is that because of the small sample size and objective responses with regards to the usability testing portion of this study only one reviewer (JR) evaluated responses and interrater and/or intrarater reliability was not needed [39]. Also because this was not an exhaustive qualitative review, but rather a preliminary exploration into the CHWs desires and needs with regards to data entry, only one reviewer (JR) extracted quotes from his notes and observations of the focus group.

Further limitations to validity are also present. The conclusions and internal validity could have been affected by CHWs acting as patients instead of utilizing actual patients for CHWs to interview. We believe that this limitation is mitigated by the fact that clinic physicians created the vignettes and have an intimate understanding of the interactions that CHWs have with patients. Construct validity may have been affected as all of the data fields on the paper forms routinely utilized by the CHWs were not present in the mobile application which could have affected their normal workflow. The purpose of this study, however, was to gain insight into the issues that may arise for CHWs when using a mobile application as a form in order to design a more optimal mobile application.

Much recent mHealth literature has drawn attention to the fact that the evidence base for mHealth approaches improving health outcomes is lacking [40, 41]. There is an increasing recognition of the importance of studying implementation/process as a way to achieve sustained improvements in health outcomes [12, 17, 18, 42–44]. While a number of mHealth initiatives have been undertaken or are ongoing in Brazil, these have mostly focused on data collection or on medical management [45, 46]. Even the e-SUS initiative, while impressive, appears to be largely focused on informatization of the existing system in order to improve SUS administration. Our study, by focusing on usability, as measured by data entry accuracy and end-user satisfaction, is thus an important contribution to the body of mHealth literature in Brazil. Our future plans include building and field-testing the prototype app developed here, with the goal of further understanding local process. The insights gained through this process will be a crucial part of building a system that will both serve its users (and thus be utilized) and, in doing so, have a positive impact on community health. The iterative process we envision, optimizing the usability of a system, is likely to be an integral part of the sustained success of any mHealth initiative.

## Conclusions

Mobile health is among the most rapidly growing sectors in healthcare. Numerous initiatives have been undertaken or are underway in a global health context. We show here that in a small cohort of community healthcare workers in a low middle income country (Brazil), that there were few technological barriers to the adoption of a mobile health app to replace paper and pencil forms. In contrast, we did identify usability barriers relating to dissatisfaction with existing work processes and concerns about security. These results highlight the importance of deeply understanding local process prior to implementing any high-technology solution. Doing so is likely to be vital to the success of any global mHealth initiative.

## Appendix 1

### Clinical Vignettes

The vignettes used to inform the data entry exercise are listed in Portuguese and English.

#### Paciente 1

Dona Maria dos Santos, 54 anos, natural e procedente de São Paulo, doméstica, casada, com 3 filhos. Mora próximo à UBS com os 3 filhos e marido em casa de alvenaria, com coleta de lixo e esgoto encanado. Nega tabaco, nega uso de bebida alcoólica, nega conhecimento de doença prévia. Não tem convenio.

#### Patient 1

Mrs Maria dos Santos, 54 years old, São Paulo native, maid, married, with 3 children. Lives near the UBS (the health center) with her 3 children and husband in a brick home, with trash collection, and closed (piped) sewage. Denies smoking, denies drinking, denies any prior medical conditions. Does not have health insurance.

#### Paciente 2

Dona Roberta de Moraes, 51 anos, natural do Rio de Janeiro e mora atualmente próximo à UBS, doméstica, casada, com 2 filhos. Mora com os 2 filhos e marido em casa de alvenaria, com coleta de lixo e esgoto encanado. Fuma 1 maço ao dia, nega uso de bebida alcoólica, tem Hipertensão arterial há 5 anos em uso de captopril 50 mg cada 12 hs. Não tem convenio.

#### Patient 2

Mrs. Roberta de Moraes, 51 years old, from Rio de Janeiro and lives close to UBS (health center), maid, married with two children. Lives with her 2 children and husband in a brick home, with trash collection and closed (piped) sewage. Smokes 1 pack of cigarettes a dat, denies alcohol use, has had hypertension for 5 years and takes captopril 50mg every 12h. Does not have health insurance.

#### Paciente 3

Dona Elaine Soares, 65 anos, natural da Bahia e mora atualmente próximo à UBS, doméstica, casada, com 1 filho. Mora com marido em casa de alvenaria, com coleta de lixo e esgoto encanado. Fuma 1 maço ao dia, nega uso de bebida alcoólica, tem Diabetes há 5 anos em uso de insulina e Hipertensão arterial há 5 anos em uso de captopril. Não tem convenio.

#### Patient 3

Mrs. Elaine Soares, 65 years old, from Bahia and lives close to the health center, maid, married with one child. Lives with her husband in a brick home, with trash collection and closed sewage. Smokes one pack per dat, denies drinking alcohol, has had diabetes for 5 years, uses insulin. Also has high blood pressure for which she takes captopril. Does not have health insurance.

## Abbreviations

BRIC: Brazil, Russia, India, China
CHW: Community Health Worker
CSEBF: Centro de Saúde Escola Barra Funda
ESF: Equipe de Saúde da Família
iOS: Apple mobile operating system
mHealth: Mobile health applications
SUS: Sistema Único de Saúde
UCSF: University of California San Francisco
